# Transactional Evaluation of the Influence of Diet Consistency on Transverse Maxillary Deficiency, Plaque Index and Dental Caries in Pediatric Patients: A Cross-Sectional Study

**DOI:** 10.3390/nu17060982

**Published:** 2025-03-11

**Authors:** Alessandro Ugolini, Alessandro Bruni, Andrea Abate, Alessandro Chiesa, Serena Bellesia, Valentina Lanteri

**Affiliations:** 1 Department of Sciences Integrated Surgical and Diagnostic, University of Genova, 16126 Genoa, Italy; 2 Surgical, Medical and Dental Department, University of Modena and Reggio Emilia, 41121 Modena, Italy; alebruni@unimore.it (A.B.); alessandro.chiesta@unimore.it (A.C.); serebellesia@gmail.com (S.B.); valentina.lanteri@unimore.it (V.L.)

**Keywords:** diet consistency, malocclusion, maxillary hypoplasia, dental hygiene

## Abstract

Objectives: This study examines the association between a predominantly soft-textured diet and clinical signs of maxillary bone hypoplasia, such as maxillary constriction and related malocclusions like crossbite. A secondary aim is to assess whether this diet correlates with dental caries and higher plaque index in children with early mixed dentition. Methods: A total of 106 pediatric patients (4–12 years) were enrolled during routine visits (June 2022–February 2024), divided into 53 “cases” (maxillary hypoplasia and malocclusions) and 53 “controls” (normal maxillary development, no malocclusions). Patients with congenital craniofacial malformations were excluded. Dietary habits were assessed using a food questionnaire categorizing foods into four consistencies (“Semi-Liquid”, “Creamy”, “Soft”, and “Solid”). The DMFT index was calculated, considering only missing teeth due to caries. The plaque index (PI) was recorded at the first visit to evaluate the relationship between food texture and plaque accumulation. Statistical analyses included Student’s *t*-test, Z-test, Chi-square test, and Fisher’s exact test. Results: Mixed breastfeeding was common in both groups with no significant difference. However, natural breastfeeding was significantly more frequent in the non-crossbite group. A high-arched palate was more prevalent in the crossbite group (*p* = 0.042 *). Soft food consumption was significantly higher in the crossbite group compared to controls (*p* = 0.032 *). A statistically significant association was found between caries prevalence (DMFT > 0) and posterior crossbite (*p* = 0.04). Furthermore, the relationship between the dental plaque index and food consistency demonstrated a statistically significant result. In particular, there was a correlation between soft food and semi-solid foods and the plaque index (χ^2^ = 3.55, *p* = 0.04). Conclusions: Posterior crossbite is associated with increased consumption of soft foods, potentially reducing the mechanical stimulation essential for maxillary growth. Conversely, non-crossbite subjects consume more hard foods and are more frequently breastfed naturally, reinforcing their role in craniofacial development. Additionally, posterior crossbite is associated with higher caries prevalence, indicating a potential connection between occlusion and oral health. Dietary texture also influences oral hygiene, with soft and semi-solid foods correlating with increased plaque accumulation, while no association was found with solid or hard foods.

## 1. Introduction

Transverse maxillary deficiency and dental caries represent two of the most prevalent oral health challenges in pediatric populations worldwide, both deeply influenced by modern lifestyle and dietary habits [1]. Transverse maxillary deficiency, often characterized by unilateral or bilateral posterior crossbite, affects up to 33% of individuals, with variations among ethnic groups [2]. This condition is closely linked to the reduced intensity and duration of mastication observed in postindustrial urban populations. Modern diets, dominated by soft and processed foods, fail to provide the mechanical stimulation required to promote maxillary growth [3,4]. Insufficient chewing effort leads to inadequate strain on the maxillary bone, impeding its development [4]. Additionally, pathological oral breathing, whether due to nasopharyngeal obstruction or habitual patterns, further exacerbates maxillary underdevelopment by disrupting muscular forces and reducing nasal airway use, which are essential for proper bone stimulation [5,6]. In contrast, historical populations consuming fibrous, unprocessed foods engaged in prolonged and intensive mastication, promoting natural forces that stimulated maxillary expansion and optimal skeletal growth [7].

Research demonstrates that the decreased masticatory demands of modern diets contribute to a rise in malocclusions such as posterior crossbites and maxillary hypoplasia [8,9]. Studies comparing rural and urban pediatric populations confirm that children with diets requiring less chewing effort exhibit higher incidences of transverse maxillary deficiencies and associated malocclusions [7,10,11]. This evidence highlights the pivotal role of dietary consistency in skeletal development, emphasizing that reduced masticatory forces, coupled with altered breathing patterns, are critical etiological factors in maxillary deficiency [12,13]. Despite recent declines in caries prevalence in developed countries over the past three decades, the disease persists as a widespread issue [14,15]. Its etiology is multifactorial, involving host factors such as saliva composition and enamel characteristics, oral microflora, and substrate-related factors like dietary habits and oral hygiene. Diets rich in sugars and processed foods exacerbate the risk of enamel demineralization, plaque accumulation, and caries formation, while diets containing protective components, such as calcium-rich dairy products, help maintain enamel integrity by promoting remineralization [16,17].

Dental crowding, a common occlusal characteristic associated with malocclusions such as maxillary hypoplasia, further complicates the picture [18]. Crowding alters interproximal tooth contacts, creating improper embrasures that facilitate food retention and plaque accumulation, increasing the risk of caries. Previous studies have reported conflicting findings regarding the relationship between malocclusions and dental caries, with some indicating a positive correlation and others showing no or even negative associations [18,19]. This inconsistency underscores the complexity of the relationship and the need for further investigation.

The interplay between dietary consistency, skeletal development, and oral health is a crucial area of study [20]. Modern dietary patterns have not only reduced the masticatory forces required for optimal maxillary development but have also contributed to an increased prevalence of dental caries through poor oral hygiene and increased plaque retention [20]. Despite significant research on these individual factors, no study has yet simultaneously analyzed the relationships among dietary texture, transverse maxillary deficiency, plaque index, and dental caries. Currently, there is insufficient scientific evidence regarding the role of dietary consistency in the development of maxillary bones, musculature, teeth, and soft tissues that form the stomatognathic system.

Research focused on this topic could highlight an association between diet and dental and skeletal development. These results may lay the groundwork for future clinical studies aimed at further investigating the potential role of diet as a factor capable of influencing occlusion and facial skeletal proportions, and/or as a variable influenced by these factors.

Therefore, the aim of this study is twofold: the primary objective is to analyze the association between a diet predominantly based on soft-textured foods and the presence of clinical features indicative of maxillary bone hypoplasia, such as maxillary constriction, mandibular retrusion, and/or mandibular hypoplasia, as well as associated dental malocclusions like crossbite. The secondary objective is to investigate the relationship between a diet predominantly based on soft-textured foods and the presence or absence of dental caries and an increased plaque index in patients with early mixed dentition. The hypothesis of this study is that a soft diet during childhood may inhibit maxillary growth, potentially leading to the onset of malocclusions and a greater presence of plaque and dental caries.

## 2. Materials and Methods

Participants were recruited during routine outpatient visits at the Orthodontics Clinic, Department of Dentistry and Maxillofacial Surgery, Azienda Ospedaliero-Universitaria di Modena, between 19 June 2022 and 5 February 2024. This study recruited patients aged 4–12, dividing them into two groups: a case group with maxillary constriction, defined by a posterior interarch transverse discrepancy of at least 5 mm, and a control group with normal maxillary development and no transverse discrepancy. Data collection included a comprehensive anamnesis and clinical examination, with measurements of transverse discrepancy, as well as baseline photographic, radiological, and orthodontic documentation.

This study was conducted following the ethical standards of the Declaration of Helsinki, and ethics approval was obtained from the institutional review board prior to recruitment. This study, identified on the SIRER platform with the code “ID 4603—Diet, Malocclusions, and Oral Respiration”, was approved by the Ethics Committee of the Emilia North Vast Area on 5 June 2023. Favorable opinions were issued through the records “Protocol AOU 0017553/23” (concerning the Pediatric Unit) and “Protocol AOU 0017554/23” (concerning the Dentistry and Oro-Maxillofacial Surgery Unit), followed by the official authorization from the University Hospital of Modena on 16 June 2023.

This research represents an interventional case–control study, which involves the selection of a sample of 53 cases and 53 controls, defined as follows: Cases: Pediatric patients diagnosed with hypoplasia of the maxillary bones (including maxillary constriction, mandibular retrusion, and/or mandibular hypoplasia) and associated dental malocclusions, such as crossbite. Controls: Pediatric patients with clinically normal development of the maxillary bones and the absence of dental malocclusions.

### 2.1. Eligibly Criteria

Participants were divided into two groups based on their clinical presentation. The case group included pediatric patients aged 4 to 12 years diagnosed with posterior crossbite, either unilateral or bilateral. In contrast, the control group comprised pediatric patients within the same age range who exhibited normal maxillary development and no evidence of unilateral or bilateral posterior crossbite.

Patients were excluded from this study if they presented with congenital craniofacial malformations, such as cleft lip or palate, or if they were considered medically vulnerable due to health conditions that exempted them from standard clinical assessments.

### 2.2. Data Collection

Data collection included a detailed anamnesis and orthodontic clinical examination, supported by baseline photographic and radiological documentation. Anamnesis covered early-life feeding practices, including type (breastfeeding or bottle-feeding) and duration, as well as oral habits such as prolonged pacifier use or thumb-sucking.

Parents also provided information on premature or trauma-related loss of deciduous or permanent teeth and any history of atypical swallowing patterns.

Orthodontic clinical examinations primarily focused on posterior crossbite, which was the key parameter for categorizing participants into case and control groups. Additional evaluations included dental relationships such as molar and canine classes. Other occlusal characteristics analyzed were open bite, deep bite, anterior crossbite, crowding, and the presence of diastema. Signs of oral breathing, including lip incompetence, low tongue posture, and high-arched palate, were documented. Parental reports on night-time breathing patterns, including habitual oral breathing, nasal breathing, or mixed oronasal breathing, were also recorded (Supplementary Materials).

Dietary habits and preferences were assessed using a parent-reported questionnaire designed to classify dietary habits into four food consistency categories (Table S1). The questionnaire, developed in collaboration with the Metabolic and Clinical Nutrition Unit, was administered during the initial evaluation. Food consistency categories were adapted from dietary standards for patients with dysphagia to ensure an objective classification of food textures.

Each food item was assigned an arbitrary score based on its consistency, ranging from 0 (liquid) to 4 (solid). Similarly, consumption frequency was assigned a score from 0 (“never/less than once a month”) to 5 (“daily”). The mean frequency of consumption for each food item was calculated, organized chronologically according to the order in which the items appeared in the dietary questionnaire. Items consumed multiple times throughout the day were recorded with differing average frequencies depending on the meal type considered.

To ensure the reliability and relevance of the dietary questionnaire, it was validated with a pilot group before its application to the study population, and all the questionnaires were evaluated by one examiner specialized in orthodontics.

This structured data collection provided a comprehensive dataset to explore the relationship between dietary habits, orthodontic characteristics, and dental decay.

No radiographic images, study models, or prior written documentation were used in this study. Before beginning the clinical examination phase, the two examiners (LP and DG) underwent a calibration process to align their methods. The evaluation concentrated on oral hygiene, specifically in the determination of the DMFT index, which accounts for decayed (D), missing (M), and filled (F) teeth [16,21]. Teeth were classified as missing in the index only if their loss was attributable to caries [15,22]. The dental plaque index (PI [23]) was measured with the Turesky Modification of the Quigley–Hein technique [24] for each patient at the first appointment in order to discriminate a relationship between food texture and the presence of a dental plaque. The index was calculated as follows: 0 = no plaque; 1 = separate flecks of plaque at the cervical margin of the tooth; 2 = a thin continuous band of plaque (up to 1 mm) at the cervical margin of the tooth; 3 = a band of plaque wider than 1 mm but covering less than one-third of the crown of the tooth.

### 2.3. Statistical Analysis and Sample Size Calculation

Descriptive statistics were performed to summarize the collected data. Categorical variables were expressed as percentages, while continuous variables were presented as means ± standard deviations. Differences between means in the two groups were assessed using Student’s *t*-test. The normality of continuous variables was verified using the Shapiro–Wilk test, and homogeneity of variances was assessed with Levene’s test.

Univariate analyses were conducted to evaluate the associations between posterior crossbite (case group) and no posterior crossbite (control group) and each clinical and anamnesis parameter. Continuous variables were analyzed using Student’s *t*-test, while categorical variables were assessed using Chi-squared tests. Fisher’s exact test was applied in cases of small sample sizes. Relative risk with 95 percent confidence intervals was calculated as a measure of association. A *p*-value of less than or equal to 0.05 was considered statistically significant for the analyses.

The reliability of the dietary questionnaire was evaluated through a test–retest analysis involving 15 participants representative of the target population. Parents completed the questionnaire on two separate occasions, spaced one week apart, and response consistency was measured using the Intra-class Correlation Coefficient (ICC). The resulting ICC value of 0.87 demonstrated excellent reliability, confirming the questionnaire’s effectiveness in assessing dietary habits concerning food texture and consumption frequency.

The sample size calculation assumed that approximately 8 percent of the pediatric population presents with posterior crossbite [25]. Based on this, it was hypothesized that a 21 percent difference would exist in dietary patterns between participants consuming predominantly crunchy and/or fibrous foods and those consuming predominantly soft-consistency foods. To detect this difference with 80 percent power (1 − β = 0.80) at a significance level of 5 percent (α = 0.05), a sample size of 53 cases and 53 controls (total *n* = 106) was required, maintaining a 1:1 case-to-control ratio.

Statistical analyses, including the sample size calculation, were conducted using STATA version 17 (StataCorp LP, College Station, TX, USA).

## 3. Results

A total of 106 participants were included in the study, allocated evenly between the crossbite group (*n* = 53) and the non-crossbite group (*n* = 53). The demographic and clinical characteristics, stratified by crossbite and non-crossbite groups, are summarized and analyzed in Table 1. No dropout or excluded subjects due to incomplete data were found.

No significant differences were observed between the groups regarding gender distribution (female: 28; male: 25; *p* > 0.05). Ethnic composition was predominantly Caucasian in both groups, with a significant overrepresentation of Asian participants in the crossbite group (*p* = 0.045 *) (Table 1).

Mixed breastfeeding was more common in both groups, with no statistically significant difference (*p* = 0.412). Natural breastfeeding was significantly more common in the non-crossbite group compared to the crossbite group (*p* = 0.032 *). Reduced masticatory efficiency was significantly more prevalent in the crossbite group compared to the non-crossbite group (*p* = 0.023 *). No significant differences were found in atypical swallowing patterns (*p* = 0.198) or the presence of bad habits or oral dysfunction (*p* = 0.167).

A significantly higher prevalence of a high-arched palate was observed in the crossbite group compared to the non-crossbite group (*p* = 0.042 *). Other morphological features, such as open bite, deep bite, anterior crossbite, crowding, and diastema, showed no statistically significant differences between the groups (*p* > 0.05).

Participants in the crossbite group had significantly higher BMI values compared to the non-crossbite group (*p* = 0.035 *). Age distribution was similar between the groups, with no significant differences (*p* = 0.345).

Dietary habits were analyzed based on food consistency categories and their association with the presence or absence of crossbite, as summarized in Table 2.

Soft food consumption was significantly higher in the crossbite group compared to the non-crossbite group (*p* = 0.032 *). This category included items such as yogurt, mashed potatoes, and gelato, which were consumed more frequently by participants with crossbite.

Semi-solid food consumption, including items such as pasta, soft bread, and cooked vegetables, was higher in the crossbite group compared to the non-crossbite group, though the difference was not statistically significant (*p* = 0.089).

Consumption of solid foods, such as crackers, raw carrots, and nuts, was higher in the non-crossbite group compared to the crossbite group, though the difference was not statistically significant (*p* = 0.115). Consumption of hard foods, including items such as hard cheeses, raw celery, and fried potatoes, was significantly higher in the non-crossbite group (*p* = 0.029 *).

A statistically significant relation between caries prevalence (DMFT >0) and posterior crossbite was found (*p* = 0.04) (Table 3).

Furthermore, the relationship between the dental plaque index and food consistency demonstrated a statistically significant result.

In particular, there was a correlation between soft food and semi-solid foods and the plaque index (χ^2^ = 3.55, *p* = 0.04) (Table 4). No significant results were found concerning the relationship between the dental plaque index and solid and hard foods (Table 4).

## 4. Discussion

The present research offers valuable insights into the relationship between dietary consistency, dental plaque, dental caries, and the presence of posterior crossbite in growing patients. The findings indicated that individuals with posterior crossbite had a significantly higher intake of soft foods compared to those without crossbite. In contrast, participants without crossbite showed a significantly greater consumption of semi-solid and hard foods. These results support the hypothesis that food texture and, consequently, masticatory function, play a key role in craniofacial development. The link between posterior crossbite and elevated BMI identified in this study indicates that dietary habits may simultaneously affect both craniofacial structure and overall nutritional status [26]. This relationship suggests that food texture and consumption patterns influence not only maxillary development but also metabolic health, underscoring the broader impact of diet on both physical growth and orofacial function.

This study emphasizes the significant influence of breastfeeding on craniofacial development and its role in preventing malocclusions, in line with existing research [27,28]. The higher incidence of exclusive breastfeeding among children without posterior crossbite supports the notion that natural breastfeeding encourages proper masticatory muscle activity and contributes to balanced orofacial development [29]. This process involves coordinated movements of the tongue, lips, and jaw, generating mechanical stimulation on the palate and dental arches [30], which helps shape wider dental arches and improve occlusal alignment. Additionally, prolonged breastfeeding beyond six months has been linked to a lower risk of developing malocclusions, including posterior crossbite, reinforcing its protective function [28,31].

The widespread practice of mixed breastfeeding observed in both groups reflects modern feeding trends influenced by cultural and socioeconomic factors [32]. While mixed feeding may still provide some developmental benefits, reliance on bottle feeding and pacifier use has been associated with reduced masticatory activity, altered swallowing patterns, and increased mouth breathing, all of which can negatively impact craniofacial growth and occlusal development [33,34].

Moreover, this study revealed a significantly higher prevalence of high-arched palates in the crossbite group compared to the non-crossbite group. This finding further supports the role of dietary texture as a crucial factor in shaping masticatory function and craniofacial development [35,36]. Participants with posterior crossbite exhibited a greater consumption of soft foods, which require less chewing effort and may fail to provide the necessary mechanical stimulation for proper maxillary growth. These results are consistent with previous studies showing that a predominantly soft diet can adversely affect craniofacial bone development during the growth period [35,37,38,39].

This finding aligns with anthropological research indicating that populations consuming harder, fibrous diets tend to develop broader dental arches and have lower rates of malocclusion [40,41]. Studies comparing dietary patterns across different cultural and socioeconomic contexts, such as non-developed versus developed populations, further support the notion that diet consistency plays a role in craniofacial development [10,42,43,44]. The hypothesis that dietary texture influences orofacial growth suggests that a diet requiring greater masticatory effort enhances chewing efficiency, which may contribute to a lower prevalence of malocclusions [36]. Maxillary expansion is a well-established orthodontic approach for the correction of transverse deficiencies in growing patients [45]. This objective can be achieved through various orthodontic appliances, which differ in their biomechanical properties and in the extent to which they induce skeletal or dentoalveolar expansion [46,47,48]. By increasing maxillary width, expansion facilitates proper occlusal development and may mitigate the effects of reduced masticatory function associated with a predominantly soft diet. Given the role of mastication in craniofacial growth, future research should explore whether a combined approach integrating dietary modifications and early orthodontic intervention could further optimize maxillary development.

A statistically significant association was observed between caries prevalence (DMFT > 0) and posterior crossbite. The study also examined the hypothesis that certain occlusal traits might contribute to an increased risk of dental caries. Among the occlusal characteristics analyzed, only crossbite showed a significant correlation with a higher prevalence of caries, possibly due to its link with dental crowding [13,49]. Additionally, this may also be attributed to a diet primarily composed of liquid foods, which, beyond contributing to maxillary hypoplasia and the presence of crossbite as previously demonstrated, could also lead to a higher prevalence of caries.

This consideration is further supported by the findings of the present study, in which the relationship between the dental plaque index and food consistency demonstrated a statistically significant result. Specifically, a correlation was observed between the consumption of soft and semi-solid foods and a higher plaque index. Conversely, no significant results were found regarding the relationship between the dental plaque index and the intake of solid and hard foods.

## 5. Limitations

This study presents limitations inherent to its case–control design. Although measures were taken to minimize recall bias, relying on parent-reported dietary questionnaires may have led to inaccuracies, especially regarding the texture and frequency of food intake.

The participant pool was predominantly composed of Caucasian individuals, reflecting the demographic characteristics of the Italian population where the study was conducted. While this aligns with findings from previous European research, it may restrict the applicability of results to populations with different genetic backgrounds and environmental influences. Dietary habits, particularly the consumption of fibrous versus processed foods, vary across ethnic groups and are key factors influencing dental arch development and malocclusion prevalence.

To improve generalizability, future research should consider a broader range of ethnic and cultural backgrounds.

Furthermore, this study did not control for additional variables that could impact craniofacial development, such as genetic predisposition, systemic health conditions, or non-nutritive sucking behaviors. To strengthen the validity of these findings, future studies should adopt longitudinal designs, include larger and more diverse populations, include other craniofacial variables, such as facial divergence that could be influenced by the diet consistency, and employ more advanced methodologies to explore these relationships and their clinical significance in greater depth.

## 6. Conclusions

- Participants with posterior crossbite tend to consume significantly more soft foods, which require minimal chewing effort, potentially limiting the mechanical stimulation essential for optimal maxillary growth.
- Non-crossbite participants demonstrate a higher intake of hard foods, supporting the hypothesis that a diet rich in firmer textures contributes to stronger craniofacial development and improved masticatory function.
- Natural breastfeeding is more prevalent among non-crossbite participants, further reinforcing its crucial role in promoting healthy craniofacial development through enhanced muscle activity and mechanical stimulation.
- Patients with posterior crossbite also exhibit a higher prevalence of dental caries, suggesting a possible link between occlusal characteristics and oral health.
- Dietary texture appears to influence oral hygiene status, as a significant association was found between food consistency and plaque accumulation. Participants consuming predominantly soft and semi-solid foods showed higher plaque index values, whereas no significant relationship was observed between plaque index and the consumption of solid or hard foods.

These findings emphasize the importance of dietary texture and feeding practices in craniofacial development. A diet rich in harder, fibrous foods and exclusive breastfeeding during early life could serve as preventive strategies for malocclusions, potentially reducing the need for orthodontic treatments.

## Figures and Tables

**Table 1:** Demographic and clinical characteristics of patients with and without crossbite.

Gender and Ethnicity	Total	No Crossbite	Crossbite	*p*-Value
Gender: Female	50	28	22	0.456
Gender: Male	56	25	31	0.345
Ethnicity: African	10	6	4	0.567
Ethnicity: Caucasian	86	45	41	0.213
Ethnicity: Asian	10	2	8	0.045 *
**Type and Duration of Breastfeeding**	**Total**	**No Crossbite**	**Crossbite**	***p*** **-Value**
Breastfeeding type: Mixed	60	21	39	0.045 *
Breastfeeding type: Natural	46	19	27	0.412
Breastfeeding duration (Mean ± SD)	17.8 ± 9.4	19.9 ± 9.4	16.2 ± 10.4	0.150
**Functional Alterations**	**Total**	**No Crossbite**	**Crossbite**	***p*** **-Value**
Atypical swallowing	28	12	16	0.198
Harmful habits	34	15	19	0.167
Premature loss of teeth	20	10	10	0.345
Reduced masticatory efficiency	30	8	22	0.023 *
**Morphological Features**	**Total**	**No Crossbite**	**Crossbite**	***p*** **-Value**
Open bite	15	9	6	0.478
Deep bite	10	6	4	0.654
Anterior crossbite	40	24	16	0.389
Gothic palate	50	30	20	0.042 *
Dental crowding	45	25	20	0.333
Diastema	20	10	10	0.567
**Continuous Parameters**	**Total**	**No Crossbite**	**Crossbite**	***p*** **-Value**
Age (Mean ± SD)	8.5 ± 2.0	8.7 ± 2.1	8.3 ± 2.1	0.345
BMI (Mean ± SD)	22.1 ± 6.1	19.5 ± 6.7	23.8 ± 6.7	0.035 *

* Statistically significant results.

**Table 2:** Dietary patterns by food consistency in crossbite and non-crossbite groups and different DMFT index values.

Food Consistency	Total (*n* = 106)	Non-Crossbite (*n* = 53)	Crossbite (*n* = 53)	*p*-Value	DMFT = 0%	DMFT > 0%	*p*-Value
Soft Foods	3.5 ± 1.8	3.2 ± 1.7	3.9 ± 1.9	0.045 *	39.8%	60.2%	0.049 *
Semi-Solid Foods	2.2 ± 1.5	2.0 ± 1.4	2.5 ± 1.6	0.089	22.6%	71.4%	0.022 *
Solid Foods	1.8 ± 1.7	2.0 ± 1.6	1.5 ± 1.8	0.215	52.5%	47.5%	0.12
Hard Foods	0.9 ± 0.8	1.0 ± 0.7	0.8 ± 0.9	0.342	46.2%	53.8%	0.091

* Statistically significant results.

**Table 3:** Caries prevalence (DMFT > 0) and posterior crossbite relationship.

Variable	DMFT = 0%	DMFT > 0%	χ^2^ for Trend
Posterior Cross Bite	38.3%	61.7%	χ^2^ for trend = 3.96; *p* = 0.04 *

* Statistically significant results.

**Table 4:** Dental plaque index and food consistency relationship.

Food Consistency	Plaque Index	χ^2^ for Trend
Soft Foods	3.1 ± 1.8	χ^2^ for trend = 3.55; *p* = 0.04 *
Semi-Solid Foods	3.5 ± 1.5	
Solid Foods	1.8 ± 1.7	*p* = 0.08
Hard Foods	0.9 ± 0.8	*p* = 0.12

* Statistically significant results.

## Data Availability

The data presented in this study are available on request from the corresponding author due to (specify the reason for the restriction).

## Supplementary Materials

The following supporting information can be downloaded at: https://www.mdpi.com/article/10.3390/nu17060982/s1, Table S1: Food consistency categories.
