# Supplementary material for: Transactional Evaluation of the Influence of Diet Consistency on Transverse Maxillary Deficiency, Plaque Index and Dental Caries in Pediatric Patients: A Cross-Sectional Study
Source: Nutrients. 2025 Mar 11;17(6):982. doi: 10.3390/nu17060982 (PMC11945532; doi:10.3390/nu17060982)
Supplement: Supplementary file 1 [file nutrients-17-00982-s001.zip › nutrients-3520113-supplementary.pdf]

Supplementary Materials:

Table S1. FOOD CONSISTENCY CATEGORIES

1. "LIQUID" CATEGORY

- Water
- Tea
- Carbonated drinks

---

2. "SEMI-LIQUID" CONSISTENCY (HOMOGENIZED FOODS)

- Juice and/or fruit smoothies
- Milk
- Milkshakes
- Pudding
- Custard and/or Nutella
- Fresh and creamy cheeses (e.g., Philadelphia) or aged cheeses as long as they are grated
- Pasta and/or rice soups in broth/puree
- Sauces (e.g., mayonnaise, tuna sauce)

---

3. "CREAMY" CONSISTENCY (PUREED FOODS)

- Jam
- Scooped ice cream (without wafer/biscuit/coating)
- Yogurt (plain, creamy, and/or with fruit or other pieces)
- Polenta
- Boiled potatoes
- Mashed potatoes
- Pureed vegetables and/or legumes
- Cereal creams (semolina)

---

4. "SOFT" CONSISTENCY (i.e., “SEMI-SOLID” AND “SOFT SOLID” FOODS, NOT BLENDED, BUT CHOPPED OR COOKED AND CHOPPED; a combination of foods for grade 3 and 4 dysphagia):

- Rice (and other grain cereals like barley, quinoa, couscous…)
- Cooked, non-stringy meats (e.g., chicken, turkey, bresaola…)
- Cooked ham (and other cooked deli meats like mortadella)
- Soft bread (like sandwich bread) and/or *gnocco* (a type of flatbread)
- Cooked vegetables (non-fibrous), including minestrone soup
- Bananas, figs, and cooked fruits (like stewed apples)
- Whole legumes (including legume soup), excluding chickpeas
- Dry pasta and/or baked pasta (e.g., lasagna)
- Potato gnocchi (a chewy and sticky food that adheres to the palate)
- Pizza (margherita and/or with toppings)
- Savory pies (e.g., *erbazzone* [a traditional Italian savory pie, no direct translation available])
- Snacks, brioches, and soft breakfast cakes (e.g., pound cake)

- Ice cream with wafer (cone)/biscuit/coating (such as certain packaged ice creams)
- Eggs (omelets, fried, and/or boiled)
- Stringy cheeses (e.g., mozzarella)
- Fish flesh (cooked fish free of head, tail, bones, and skin) and/or raw fish (e.g., sushi)
- Canned tuna

---

5. "SOLID" CONSISTENCY (UNPROCESSED FOODS)

This category was created by us to group foods not recommended for grade 4 dysphagia (the least severe form). It includes unprocessed foods, particularly DRY, CRUMBLY, and CRUNCHY items or FIBROUS and STRINGY foods:

- Baked goods such as crackers, flatbreads, breadsticks, chips, crunchy bread (like baguette), rusks, biscuits
- Cakes with a crunchy dough and/or filling (e.g., *sbrisolona* [a traditional Italian dessert, no direct translation available])
- Industrial cereals (e.g., Kellogg’s and muesli)
- Nuts
- Fresh fruit (not necessarily peeled, deseeded, or cored, and consumed whole), except bananas, figs, and cooked fruit (like stewed apples)
- Raw vegetables (especially overly fibrous ones such as carrots, celery, fennel, spinach, artichokes)
- Oven-baked or fried potatoes
- Aged cheeses
- Chickpeas (which have a firmer texture compared to other legumes)
- Stringy meats, especially raw (e.g., carpaccio)
- Raw ham (and other raw cured meats like salami, speck, pancetta)
- Fish such as mollusks (squid, octopus… due to their chewy texture) and/or crustaceans (shrimp, prawns… where removing the head, tail, bones, and skin is challenging), fried seafood mixes
- Candies (chewy sweets, licorice…) and/or chocolates
